# Predictors of Suicide Ideation and Attempt Planning in a Large Sample of New Zealand Help-Seekers

**DOI:** 10.3389/fpsyt.2022.794775

**Published:** 2022-02-25

**Authors:** Daniel Shepherd, Stephen Taylor, Rita Csako, An-Tse Liao, Renee Duncan

**Affiliations:** ^1^Department of Psychology, Auckland University of Technology, Auckland, New Zealand; ^2^School of Psychology, University of Auckland, Auckland, New Zealand

**Keywords:** suicide, suicide ideation, suicide planning, risk factors, protective factors

## Abstract

**Aim:**

Suicide is a major public health concern worldwide. The present study explores the risk factors for suicide ideation and suicide attempt planning by analyzing anonymized data collected by a New Zealand telephone helpline.

**Method:**

A nation-wide helpline, Lifeline Aoteroa, provided data from distressed callers obtained from May 2017 to April 2018. The analyzed sample consisted of 32,889 counseling calls of clients with a wide range of presenting issues. Frequency analysis and multivariable logistic regression were adopted to determine risk and protective factors associated with two types of suicide behaviors: suicide ideation and suicide planning.

**Results:**

Risk factors for suicide ideation and suicide planning included: hopelessness, sadness, fear, not coping with symptoms of mental health issues, mild/moderate severity self-harm, severe self-harm, urge to self-harm, alcohol and/or drug addiction, suicide in family, sexual domestic abuse. The adjusted odds rations for these risk factors ranging from 1.31 to 16.42. Protective factors included feelings of hope or joy, the opportunity to talk and, unexpectedly, feeling anxious or stuck. The adjusted odds ratios for protective factors ranged from 0.15 to 0.75.

**Conclusion:**

Risk factors were identified for both suicide ideation and for suicide attempt planning. While some of these risk factors have been reported in existing literature, there are also risk factors unique to the present study that could inform and improve suicide-screening procedures administered by clinicians or helplines.

## Introduction

The World Health Organization estimates that somewhere in the world a person willfully takes their life every 40 s (1), accounting for 1.4% of all deaths globally. In New Zealand, the prevalence of suicide is estimated to be 11 deaths per 100,000 people, though for Māori this rate is higher at around 24 deaths per 100,000 people (2). Suicide ideation involves thoughts about willingly performing self-harm with the intent to die, whereas attempt planning involves the consideration of methods as to how best to take one's life, and how to implement them (3). Suicide ideation is a common precursor to suicide and is highly correlated with suicide attempts (4), which in turn are potent risk factors for an eventual suicide death (5).

International studies have probed risk factors of suicide behavior. Non-suicidal self-injury (hereon self-harm) has been linked to suicide ideation, plans, and attempts (6). Suicide ideation and suicide behaviors have been linked to a history of intimate partner abuse or childhood abuse (7), with the latter relationship mediated by mood and anxiety disorders (8). A meta-analysis found that hopelessness, or negative expectations about the future, was a prominent risk factor for suicide ideation, suicide attempts, and suicide completion (9). In relation to mental health, the presence of a mental disorder substantially heightens the risk of suicidal behaviors, with psychiatric comorbidity significantly increasing this risk (10). Higher rates of suicidal ideation has also been noted on those with chronic pain (11) or a terminal illness (12).

In the New Zealand context, depression, anxiety, substance abuse, relationship stress, and financial problems were reported to be risk factors for suicide among farmers, as well as the general population (13). Further, the lifetime prevalence of suicide ideation is estimated to be 15.7%, and to be 5.5% for suicide planning, with a 12-month prevalence of 3.2 and 1%, respectively (14). Echoing international findings, a strong association between self-harm and suicide ideation has been reported in New Zealand (15). A study undertaken with New Zealand women revealed that those who had experienced sexual and/or physical intimate partner abuse reported high rates of suicide ideation than those who had not (16). Additionally, a sample of New Zealand HIV-positive residents indicated increased rates of suicide ideation, suicide attempts, as well as completed suicide (17). Within the New Zealand adolescent population, depression, having family or friends who have committed suicide, economic deprivation, substance abuse, sexual orientation, and being female have been identified as risk factors of suicide attempts (18, 19).

Between 50 and 80 percent of people living in New Zealand will experience problems with mental health or addiction at some point in their life, including suicide behaviors (20). Mental health services exist to improve service users' state of mind and in doing so eliminate suicide behaviors in their clients. However, uptake of services can be thwarted by factors associated with both the client (e.g., fear of stigma or denial of a problem) and the service itself (e.g., long waiting times or cost). Telephone counseling gives clients anonymity and in most instances immediate assistance, making it a more attractive option for support. There is evidence to suggest helplines can improve a service user's mental state, and more importantly, be a preventative measure for suicide by reducing the service user's suicide behavior (21). Lifeline, which operates in both Australia and New Zealand, is a telephone counseling service with a primary mission of suicide prevention (22). Lifeline Aoteroa is a New Zealand based helpline that receives hundreds of calls per day, with ~8% of those callers judged to be at risk of making a suicide attempt (23).

The present exploratory study consists of a secondary analysis of data collected by Lifeline Aoteroa from May 2017 to April 2018. The main objective was to elucidate the risk factors for suicide ideation and suicide attempt planning, and determine which risk factors are the most influential. As the present study involves callers who were alive at the time of the call, the focus was on finding the risk factors for suicide behaviors rather than for suicide *per se*. The underlying logic motivating the analysis is that as those who engage in suicide ideation and suicide planning are more likely to attempt suicide, then it is important to understand the risk factors associated with suicide ideation and planning.

## Methods

### Participants

The data came from 51,328 calls received by Lifeline from May 2017 to April 2018. The present study focused on all counseling calls (*n* = 32,889), discarding non-counseling calls, which including hang-ups, general inquiries, hoaxes, and silent calls. As the purpose of Lifeline's service is to help individuals in distress and ensure the confidentiality of the callers' identity, demographic variables that were not the focus of counseling services such as gender, ethnicity, and age, were not collected as they could potentially identify the caller. As such, because the data collected by Lifeline was not intended for research purposes, this study can be considered a secondary analysis of data. Lifeline has a set of ethical mandates protecting the privacy of their callers by anonymizing all data and explaining confidentiality if requested. This ensures the privacy of callers when the data are used for research purposes. This study was approved by the Author's institutional human research ethics committee (AUTEC 19/24).

### Data Acquisition

Categorical data was collected from Lifeline counselors and trained volunteers who, following a counseling call, used an online client management system to complete a session note. This entailed using check boxes to document the presenting issues identified during the call, coded as either “0” (not present in call) or “1” (present in call). As part of each call a counselor serially presented a prescribed list of issues (*re*: Supplementary Table 1), such that callers were all screened for the same issues. Presenting issues were clustered into the following categories: addiction; anxiety/depression (self-report); bullying; child abuse; domestic abuse and violence; employment-related; key feelings; loneliness; mental health; parenting related; physical health; mental health (clinical diagnosis); poverty/financial concerns; relationship difficulty; self-harm; sexual health; suicide, and; violence. These categories and embedded presenting issues can be viewed Lifeline's data dictionary (see Supplementary Table 1).

### Data Analysis

The data analysis was conducted using the statistical computing software “R” (24). An initial descriptive analysis was conducted to document the frequency of each presenting issue. For the purpose of this study the two dependent variables of interest were defined as the presence of suicide ideation or suicide attempt planning (both coded as 0 = No, 1 = Yes). For each presenting issue, frequencies were cross-tabulated for suicide ideation and suicide planning.

A logistic multivariable regression model was fitted for each of the two suicide variables, with a subset of the remaining categorical variables designated independent variables. A forward stepwise regression, starting with an empty model, determined the best fitting models based on the Bayesian Information Criterion (BIC). For each such selected independent variable, this approach permitted the estimations of adjusted odds ratios (AOR), which account for the effects due to all the additional variables included in the model. Given the large sample size (*N* = 32889), the large number of independent variables, and the secondary analysis nature of the study, a conservative level of statistical significance was selected at *p* < 0.001.

## Results

### Descriptive Analyses

From the whole sample of 32,889 counseling calls, there were 2,622 responses that confirmed suicide ideation (7.97%), and 704 responses that confirmed suicide attempt planning (2.14%). Supplementary Table 2 shows the frequency counts for each presenting issue for the entire sample and also separately for the suicide ideation and suicide planning variables. Scrutiny of this data reveals that, for the entire sample, the presenting issues most endorsed occur in the Key Feelings and Relationship Difficulties categories. Inspection of the cross tabulated frequencies suggest that for suicide ideation the prevalence of presenting issues is generally greater than for the entire sample, with most endorsed being mental health issues (both self-report and diagnosed), self-harm, and exposure to the suicide of another. For suicide planning, the most endorsed presenting issues mirrored those reported for suicide ideation, albeit with generally lower prevalence's.

### Regression Analyses

Table 1 displays the results of the first binary logistic model in which suicide ideation was the dependent variable, and contains adjusted odds ratios (AOR) accompanied by confidence intervals which should be consulted by the reader. Only presenting issues with changes in BIC <0 and a significant AOR are presented. The presenting issues are ranked by decreasing AOR. The effect of a presenting issue on a suicide variable can be categorized as a “risk” (AOR > 1) or a “protective” (AOR < 1) factor. A risk factor suggests that the presenting issue is positively associated with a suicide behavior, even when taking into account the effects of all other variables in the model. Similarly, a protective factor suggests that the presenting issue is negatively associated to the suicide behavior. Scrutiny of Table 1 indicates that the three self-harm behaviors, struggling with a mental illness, and knowledge of other's suicide are the dominant risk factors for suicidal ideation. Additionally, experiencing sexual abuse in a relationship or having addiction issues are also predictors of suicide ideation. Four of the 22 presenting issues were identified as protective factors by the model. These were feeling hopeful, feeling happy, ringing for a general chat, and feeling anxious. The global fitness for the suicide ideation model was estimated using the area under the curve (AUC) derived from the receiver operation characteristic (ROC), and was AUC = 0.837.

**Table 1:** Adjusted odds ratio (AOR) accompanied by confidence intervals (CI) for presenting issues predicting suicide ideation with Bayesian Information Criterion (BIC) differences below 0.

**Presenting issue**	**AOR**	**(CI 95%)**
Severe self-harm: needs medical care	6.35	(2.69, 14.9)^*^
Not coping with anxiety/depression–severe impact on QoL (Self-reported)	6.26	(5.20, 7.54)^*^
Self-harmed–mild/moderate injury	5.67	(4.12, 7.79)^*^
Not coping with mental illness–severe impact on QoL (clinical diagnosis)	4.53	(3.83, 5.36)^*^
Urge to self-harm–needing support	3.48	(2.73, 4.43)^*^
Suicide in family	3.47	(2.33, 5.16)^*^
Suicide in peer	3.25	(2.01, 5.27)^*^
Feeling hopeless	3.22	(2.87, 3.61)^*^
Struggling to manage anxiety/depression symptoms–moderate impact on QoL (Self-reported)	2.79	(2.45, 3.18)^*^
Sexual abuse from a partner	2.20	(1.65, 2.92)^*^
Issues with alcohol and/or drugs	2.10	(1.69, 2.61)^*^
Struggling to manage mental illness–moderate impact on QoL (Clinical diagnosis)	2.09	(1.80, 2.42)^*^
Feeling sad	2.05	(1.86, 2.26)^*^
Relationship breakup	1.71	(1.44, 2.03)^*^
Difficulty finding work	1.69	(1.29, 2.23)^*^
Seeking info/support	1.52	(1.20, 1.95)^*^
Feeling fear	1.51	(1.35, 1.69)^*^
Feeling guilt	1.31	(1.13, 1.51)^*^
Feeling anxious	0.75	(0.66, 0.85)^*^
Feeling hopeful	0.67	(0.55, 0.81)^*^
General chat	0.54	(0.47, 0.62)^*^
Feeling happy	0.15	(0.06, 0.36)^*^

* *p < 0.001; QOL, Quality of Life*.

*Note that AORs above 1 are risk factors, while <1 indicates a protective factor*.

Turning now to the second regression model in which suicide planning was assigned as the dependent variable (*re*: Table 2), it is noteworthy that the pattern of significance does not perfectly replicate that presented in Table 1 for suicide ideation. While severe self-harm retained the top spot as the presenting factor with the highest AOR, the second highest was feeling hopeless. In addition to the four protective factors identified in the first model, feeling stuck and self-managing symptoms with support were also protective factors. The AUC for the attempt planning model was 0.853.

**Table 2:** Adjusted odds ratio (AOR) accompanied by confidence intervals (CI) for presenting issues predicting suicide planning with Bayesian Information Criterion (BIC) differences below 0.

**Presenting issue**	**AOR**	**(CI 95%)**
Severe self-harm: needs medical care	16.42	(6.71, 40.18)^*^
Feeling hopeless	6.12	(5.04, 7.42)^*^
Suicide in family	4.17	(2.42, 7.21)^*^
Self-harmed–mild/moderate injury	4.03	(2.69, 6.05)^*^
Not coping with mental illness–severe impact on QoL (clinical diagnosis)	3.77	(2.95, 4.81)^*^
Not coping with anxiety/depression–severe impact on QoL (Self-reported)	3.58	(2.78, 4.61)^*^
Feeling numb	2.77	(1.93, 3.96)^*^
Sexual abuse	2.31	(1.47, 3.62)^*^
Issues with care provider/health professional	2.08	(1.35, 3.21)^*^
Issues with alcohol and/or drugs	1.90	(1.33, 2.72)^*^
Feeling sad	1.75	(1.47, 2.09)^*^
Feeling fear	1.53	(1.25, 1.86)^*^
Feeling emotional	1.49	(1.18, 1.88)^*^
Feeling hopeful	0.64	(0.53, 0.77)^*^
Feeling stuck	0.52	(0.40, 0.69)^*^
Feeling anxious	0.42	(0.32, 0.55)^*^
General chat	0.39	(0.29, 0.54)^*^

* *p < 0.001; QOL, Quality of Life*.

## Discussion

The current study concentrated on the relationships between presenting issues reported by Lifeline users and both suicide ideation and suicide attempt planning. Self-harm behaviors emerged as the major risk factor of suicide behaviors in the present study, specifically the act of mild/moderate severity self-harm, high severity self-harm, and for suicide ideation only, the urge to self-harm. For the current analyses, engaging in severe self-harm requiring medical attention was the most effective predictor of suicide behaviors. The intrapersonal functions of non-suicidal self-injury have been reported to regulate emotions that are strongly associated with suicidal behaviors (4). Here, self-harm acts as a coping strategy that reduces negative emotions while positive emotion such as feelings of calmness are increased (25), and thus self-harm is continually reinforced by its distress-escaping intrapersonal function. Concerningly, when stressors become overwhelming an individual may then extrapolate this distress-relief to suicide as another means of escape and, far from serving a self-preservation function, self-harm in fact may work to increase suicide behaviors (6).

Self-harm behaviors and increased suicidality are common diagnostic symptoms in many mental illnesses but prominent in Posttraumatic Stress Disorder and Borderline Personality Disorder with clear treatment recommendations including mindfulness and distress tolerance skills training based on Dialectical Behavior Therapy principles (26). The practical implication of these findings is that helpline counselors could be trained to provide brief mindfulness-based interventions to alleviate distress when identifying suicide-behavior risk, allowing the help-seeker to engage in more effective cognitive and behavioral strategies when dealing with suicidal ideation or planning.

Mental health issues, either self-reported depression/anxiety or formally diagnosed major mental illnesses, were strongly associated with suicide behaviors in the current sample. This finding is not unprecedented, with Batterham et al. (27) identifying both generalized anxiety disorder and depression as significant risk factors of suicidal ideation, with anxiety reported to be the greater risk. While the current dataset does not allow us to disentangle depression and anxiety (self-report) or types of major mental illnesses directly, the inclusion of key feelings (*re*: Supplementary Table 1) affords some reasoned interpretation of the data. Depression has repeatedly been linked to increased levels of suicide and suicide behaviors including ideation (10, 28), and especially so in the New Zealand context (14). The relationship between depression and suicide behaviors may potentially be mediated by hopelessness, specifically negative expectations of the future (29), and this proposition is reinforced within our dataset as feelings of hopelessness was likewise a significant risk factor, while feeling happy and hopeful were significant protective factors.

For those having a formal diagnosis of mental illness, issues with a care provider or health professional was a significant risk factor for suicide attempt planning, and this suggests that perceptions of formal supports is an important factor in suicide planning. This highlights the importance of publicly available helplines, such as Lifeline, as it may be perceived by the callers as available professional and social support when the assigned health-care provider is not available or the relationship between the care provider and the client is unsatisfactory. It also stresses the importance of rapport building and engaging the caller in disclosure so available and ongoing training and supervision for all helpline agents is essential to ensure the best available outcome.

Interestingly, while feelings associated with depression (i.e., feelings of hopelessness and sadness) were also significant risk factors for suicide behaviors, we found that feeling anxious was a protective factor, even though the related construct of feeling fear was a risk factor. This finding is novel and without precedence in the literature, as it is commonly reported that anxiety disorder is a risk factor for suicide behaviors [For a review see (30)]. Conceptually, anxiety is a maladaptive response to an external threat (real or imagined) or an internal conflict, while fear is the acute expression of negative emotions elicited by exposure to a real world object (31). Speculatively, our data implies that the worry of an early death may be an ongoing source of consternation, suggesting that the individual is afraid of dying and therefore less likely engage in suicide behaviors as these in themselves are threats to their survival. In contrast, feeling fear or “scared” typically relates to a specific object or event, for example, being afraid of an abusive partner. Here, fear may gave rise to either defense or escape behaviors, where for the later this may speculatively include suicide behaviors. Other arguments as to why our findings differ is that we adjusted for a greater number factors than typically reported in the literature, which is important when discerning the relationship between anxiety and suicidal behaviors [see (30), p.6]. Further, how anxiety is operationalized is also important when comparing data across studies, with most studies using multi-item inventories as opposed to the binary judgments made by the telephone counselors in the current study.

Alcohol and/or drug misuse were risk factors for suicide behavior in the current study, in line with previous studies (32) reporting that substance use disorder has is strongly associated with suicide outcomes including suicide ideation (33). In terms of alcohol, one meta-analysis reported compelling evidence that alcohol use disorder significantly increases the risk of suicidal behaviors (34). However, it is difficult to differentiate the independent effects of drugs or alcohol on suicide behaviors within the current analysis, as drugs and alcohol were merged into a single subcategory.

In contrast to Abrutyn and Mueller (35), we found that experiencing suicide in the family was a stronger risk factor for suicide ideation than experiencing one from a peer. Social Learning Theory (36) may explain the transmission of suicide behaviors between familial and non-familial social groups, whereby an individual imitates the types of suicide behaviors that they witnessed (37). This process of imitation explains the concept of “suicide contagion” or “copy-cat” suicides, especially if the person who completed the suicide has role model status (38). However, the degree to which family-related suicides induce suicide behaviors in the family members they leave behind is not so clear, and other mechanisms will likely to be operating (39). In concordance with others (40), we also found that a relationship break-up, which can cause clinical-levels of distress and depression (41), was related to suicide ideation. However, in our data a break-up was not related to suicide planning, potentially acting as a marker to differentiate the two behaviors.

Domestic abuse of the sexual kind was also identifies as a risk factor for suicide behaviors. A strong association between the two is reliably reported in the literature [e.g., (16)], albeit focusing on female victims of sexual abuse due to its greater prevalence. Sexual abuse often leads to the development of Posttraumatic Stress Disorder and suicidality is a well-established feature of this mental illness (42), often as a way of escaping the recurring distressing memories and the associated extreme psychological pain. Wolford-Clevenger et al. (43) applied Joiner's (44) interpersonal-psychological theory of suicide to explain suicide behaviors in women experiencing abuse by partners. In this scheme a number of interacting factors are implicated in suicide ideation, including self-loathing, loneliness, and hopelessness. Wolford-Clevenger et al. (43) reported that suicide ideation was related to feelings of perceived burdensomeness (i.e., self-loathing) only at high levels of hopelessness, and while we also found that hopelessness was related to suicide ideation our dataset did not have a direct measure of self-loathing to collaborate their findings.

The data affords further comparisons between the two suicide behaviors analyzed: ideation and planning. Some in the literature combine these two concepts [e.g., (34)], though the findings of others argue that the constructs are distinct both conceptually and clinically. Of relevance, Beautrais et al. (14) reported that, of their sample with a mental illness, 11.8% reported suicide ideation, but only 4.1% reported a suicide plan. While some similarities do exist within the current analysis, for example having a “general chat” with a telephone counselor was a protective factor against both behaviors, differences were also noted. For example, having a peer commit suicide was a risk factor for suicide planning but not suicide ideation, suggesting a limit to suicide contagion. Furthermore, whereas feelings of hopelessness had the 2nd largest effect on suicide planning, it was the 8th most potent risk factor for suicidal ideation, a finding that is of clinical significance and again suggesting different underlying mechanisms.

Of clinical significance was the finding that numbness was a risk factor for suicide planning but not ideation, indicating that flattened affect may be a useful marker to differentiate between those with suicide ideation who are and who are not actively planning a suicide attempt. Numbness and the feeling of emptiness often creates the state of aversive inner tension which can be alleviated by self-harm behaviors and suicide planning, as often observed in Borderline Personality Disorder (45, 46).

Another novel finding was that the feeling of being “stuck” was a significant predictor of suicide planning but not suicide ideation. In the study's context feeling “stuck” indicates indecision and not being able to move on and make changes, and speculatively that the ideation itself maybe transient. Furthermore, those reporting suicide ideation but not suicide planning maybe experiencing a moral dilemma of sorts (i.e., is it right for me to end my life?) or some other existential crisis. Alternatively they could also be experiencing sufficient levels of depression not to have the energy to progress a suicide plan. As such the use of existing cognitive-behavioral therapy approaches maybe particularly effective with those reporting suicide ideation, in as much as the patient may start to challenge dysfunctional thought processes and in doing so not progress to the planning stage (47). Pertinently, feeling stuck is a commonly observed cognitive response in depressed patients, especially in persistent depression when the patient perceives that the depression is inescapable as it is often connected to events that cannot be changed or resolved (48). It is possible that those who feel stuck might consider the option of suicide as a means of making change, and so will plan the suicide but not present with suicidal ideation.

### Clinical Implications

In summary, this research highlighted the importance of identifying suicidality when help-seekers call helplines to ensure the safety of all callers. Our data suggests that several factors can indicate suicidal risk at an early stage. These can be classified into four groups: (1) behavioral indicators, such as self-harm behaviors, alcohol and drug misuse, (2) environmental factors, such as suicide in the family, domestic abuse or violence, (3) cognitive appraisal indicators, such as hopelessness or feeling stuck, (4) emotional indicators, such as numbness or sadness. Screening for these factors can reduce the risk of suicidal behaviors and support help-seekers, the current research may inform the development of such screening tool. However, it is to be noted that only some of these can be mitigated by supportive anonymous helpline- counseling, such as the cognitive appraisal factors or the emotional indicators, whereas others, such the environmental factors or triggers are difficult to manage via phone counseling. In order to prepare helpline counselors to provide the best available support for their callers, including brief intervention strategies in their training, such as brief distress tolerance skills or emotion regulation exercises, would be highly beneficial with the aim of mitigating the impact of these (often) adverse factors when providing help via a helpline.

### Limitations

The reported analyses utilizes cross-sectional data whose primary purpose was for the management of organizational practice, which forced the focus to be on associations and not causations. As such, the analysis could not determine whether the risk factor or suicide behavior occurred first, for example, while self-harm has been found to increase the risk of suicide behaviors, it could also serve as a suicide ideation substitute (49). Following this, we were not able to assess predictive validity. Also as a result from the data originating from a service provider, the information was garnered from participants using a simplified interview schedule with binary outcomes as opposed to pre-existing and validated multi-item clinical instruments. Thus, the use of binary items representing complex constructs such as depression or anxiety instead of established multi-item psychological inventories would limit construct validity. Further, while the variable set (*re*: Supplementary Table 1) was comprehensive and allowed for a greater number of adjustments that are typically found in studies of these sorts, the categories themselves sometimes represented multiple constructs that could not be disentangled.

Another caveat is that, in the context of telephone counseling services that depend on the anonymity of both counselor and caller, the issues reported by the caller could not be independently verified, and though the organization deployed strategies to reduce “frequent callers” their presence could not be quantified. Finally, the lack of basic demographic information (e.g., gender, age, ethnicity) is a limitation, as although these factors have been definitely linked to suicide behaviors it is not known whether they would have had independent effects or changed the present analysis by adjusting the effects of other variables. Following from this, it was not possible to estimate if a sampling bias existed in the participants in terms of those who use telephone counseling over other mediums.

## Conclusion

To surmise, the present study identifies specific risk-factors for suicide ideation and attempt planning which may be of utility to suicide prevention programs, helplines, or clinics striving to prioritize at-risk groups for suicide behaviors. Arguably, suicide assessment is not a single-factor assessment process and assessments need to target not only the direct measures suicide risk, but also the broader constellation of risk factors that might play a role in expressing suicidal behaviors. As helplines are typically focused on safety planning and short-term solution-focused therapy due to their limited time with callers, the elucidation of salient risk factors may inform the development of interview plans that prioritize dominant risk factors and so become more efficient at capturing those who are high-risk. Hopes are that the findings of the current study can improve risk assessments for clinicians and helplines counselors in the pacific region.

## Data Availability Statement

The raw data supporting the conclusions of this article will be made available by the authors, without undue reservation.

## Ethics Statement

Ethical review and approval was not required for the study on human participants in accordance with the local legislation and institutional requirements. Written informed consent for participation was not required for this study in accordance with the national legislation and the institutional requirements.

## Author Contributions

DS conceived the study and assisted with data analysis. ST analyzed the data and assisted with design. RC and A-TL assisted with all aspects of the study with the exception of data analyses. RD contributed to the writing up of the manuscript and collected the data. All authors contributed to the article and approved the submitted version.

## Conflict of Interest

The authors declare that the research was conducted in the absence of any commercial or financial relationships that could be construed as a potential conflict of interest.

## Publisher's Note

All claims expressed in this article are solely those of the authors and do not necessarily represent those of their affiliated organizations, or those of the publisher, the editors and the reviewers. Any product that may be evaluated in this article, or claim that may be made by its manufacturer, is not guaranteed or endorsed by the publisher.
