# Supplementary material for: Predictors of Suicide Ideation and Attempt Planning in a Large Sample of New Zealand Help-Seekers
Source: Front Psychiatry. 2022 Feb 25;13:794775. doi: 10.3389/fpsyt.2022.794775 (PMC8913723; doi:10.3389/fpsyt.2022.794775)
Supplement: Supplementary file 1 [file Data_Sheet_1.docx]

Supplementary Material

**Supplementary Table 1 |** Self-report presenting issues of Lifeline callers.

| **Category** | **Presenting Issue** |
| --- | --- |
| **Addiction**  Issues relating to addictions. | Alcohol and/or drugs |
|  | Behavioural (e.g. internet, gaming) |
|  | Gambling |
|  | Prescription Medication |
|  | Sex/porn |
| **Anxiety/Depression**  (Self-reported)  Caller has reported experiencing anxiety and/or depression. The  Counsellor doesn’t diagnose this themselves. | Discrimination/Stigma |
|  | Issues with health provider/ professional |
|  | Issues with medication |
|  | Not coping - severe impact on QOL |
|  | Seeking info/Support |
|  | Self-managing symptoms with support |
|  | Struggling to manage symptoms |
| **Child Abuse**  The Caller has brought up child abuse during the call. This could be related to self or other, current or historical. | Emotional |
|  | Neglect |
|  | Physical |
|  | Sexual |
|  | Family Violence (witnessing) |
|  | Perpetrator |
| **Domestic Abuse and Violence**  The caller has brought up family or domestic violence. | Emotional |
|  | Physical |
|  | Sexual |
|  | Financial |
|  | Damage to property |
|  | Violence to family |
| **Employment related issues**  The caller has presented with work-related issues. | Difficulty finding work |
|  | Issues with welfare |
|  | Redundancy/unemployment |
|  | Work related stress |
| **Key Feelings**  Lists what feelings the caller presented with as assessed by the counsellor. | Anger |
|  | Anxious |
|  | Confident |
|  | Confusion |
|  | Disconnected |
|  | Elated |
|  | Emotional |
|  | Exhausted |
|  | Fear |
|  | Frustration |
|  | Grief/Loss |
|  | Guilt |
|  | Happy |
|  | Hopeful |
|  | Hopeless |
|  | Numb |
|  | Rejected |
|  | Sad |
|  | Shocked |
|  | Stressed |
|  | Stuck |
| **LGBTQI**  The caller presented with issues around gender or sexuality. | Acceptance from others |
|  | Difficulty coming out |
|  | Information |
|  | Self-acceptance |
| **Loneliness Isolation**  The caller presented as lonely | Acceptance from others |
|  | General Chat |
| **Mental Health**  (Clinical Diagnosis)  The caller explicitly talked about having a diagnosed mental illness. | Discrimination/Stigma |
|  | Issues with care provider |
|  | Issues with medication |
|  | Not coping - severe impact on QOL |
|  | Self-managing symptoms with support |
|  | Struggling to cope – moderate impact on QOL |
| **Parenting Problems**  The caller presented with parenting-related issues. | Care issues (e.g. CYFs, foster care) |
|  | Children |
|  | Co-parenting |
|  | Teens |
| **Physical Health**  The caller presents with issues related to their physical health. | Acute |
|  | Chronic |
| **Poverty/Financial concerns**  The caller presents with issues related to poverty or financial concerns. | Accommodation/refuge |
|  | Bankruptcy/debt |
|  | Food/clothing |
|  | Homelessness |
| **Relationship Difficulties**  The caller presented with relationship issues. | Issues with family |
|  | Issues with neighbours/community |
|  | Issues with partner/spouse |
|  | Issues with peers |
|  | Relationship breakup |
| **Self-harm (urge to injury)**  The caller presents with self-harm issues. | Self-harmed - mild/moderate injury |
|  | Severe self-harm – needs attention |
|  | Urge to self-harm - needing support |
| **Sexual Health**  The caller presents with issues relating to sexual health. | Abortion |
|  | Contraception |
|  | HIV/STD |
|  | Pregnancy |
|  | Puberty |
|  | Sexual Dysfunction |
| **Suicide**  The caller presented with concerns about suicide, this could be for themselves or others | Suicide attempt planned |
|  | Suicide ideation (current or historical) |
|  | Suicide in family |
|  | Suicide in peer |
| **Bullying**  Caller is concerned about bullying towards themselves or someone else. | Primarily at education provider |
|  | Primarily at the workplace |
|  | Primarily cyber bullying |
|  | Primarily SMS/TXT |
|  | Primarily street/community |
| **Violence**  The caller presented with issues relating to violence other than domestic or bullying. | Committed violent offence |
|  | Elder abuse |
|  | Harm to others expressed/planned |
|  | Intimidation/harassment (e.g stalking) |
|  | Trauma |

**Supplementary Table 2 |** Frequency counts for each presenting issue. Furthermore, each presenting issue is cross-tabulated with the frequencies for the suicide variables Ideation and Attempt.

|  | **Entire Sample** | | **Suicide Ideation** | | **Attempt Planners** | |
| --- | --- | --- | --- | --- | --- | --- |
| **Category / Presenting Issue** | **Yes** | **% Yes** | **Yes** | **% Yes** | **Yes** | **% Yes** |
| **Addiction** |  |  |  |  |  |  |
| Alcohol and/or drugs | 663 | 2.02 | 164 | 25 | 49 | 7 |
| Internet/gaming | 22 | 0.07 | 5 | 23 | 0 | 0 |
| Gambling | 43 | 0.13 | 8 | 19 | 2 | 5 |
| Medication | 30 | 0.09 | 8 | 27 | 2 | 7 |
| Sex/porn | 25 | 0.08 | 1 | 4 | 2 | 8 |
| **Anxiety/Depression (Self-reported)** | |  |  |  |  |  |
| Discrimination/Stigma | 43 | 0.13 | 20 | 47 | 9 | 21 |
| Issues with services | 124 | 0.38 | 53 | 43 | 27 | 22 |
| Issues with medication | 135 | 0.41 | 46 | 34 | 11 | 8 |
| Not coping | 676 | 2.06 | 369 | 55 | 138 | 20 |
| Seeking info/Support | 695 | 2.11 | 107 | 15 | 22 | 3 |
| Self-managing | 1549 | 4.71 | 103 | 7 | 11 | 1 |
| Struggling to manage | 1947 | 5.92 | 454 | 23 | 49 | 3 |
| **Child Abuse** |  |  |  |  |  |  |
| Emotional | 140 | 0.43 | 40 | 29 | 8 | 6 |
| Neglect | 54 | 0.16 | 15 | 28 | 2 | 4 |
| Physical | 92 | 0.28 | 23 | 25 | 7 | 8 |
| Sexual | 139 | 0.42 | 30 | 22 | 5 | 4 |
| Witness Family abuse | 48 | 0.15 | 8 | 17 | 2 | 4 |
| Perpetrator | 8 | 0.02 | 4 | 50 | 2 | 25 |
| **Domestic Abuse and Violence** | |  |  |  |  |  |
| Emotional | 927 | 2.82 | 137 | 15 | 26 | 3 |
| Physical | 396 | 1.20 | 68 | 17 | 19 | 5 |
| Sexual | 356 | 1.08 | 96 | 27 | 32 | 9 |
| Financial | 101 | 0.31 | 9 | 9 | 4 | 4 |
| Damage to property | 59 | 0.18 | 6 | 10 | 1 | 2 |
| Violence to family | 151 | 0.46 | 26 | 17 | 5 | 3 |
| **Employment related issues** | |  |  |  |  |  |
| Difficulty finding work | 505 | 1.54 | 90 | 18 | 23 | 5 |
| Issues with welfare | 167 | 0.51 | 23 | 14 | 4 | 2 |
| Redundancy/unemployment | 314 | 0.95 | 78 | 25 | 27 | 9 |
| Work related stress | 694 | 2.11 | 102 | 15 | 26 | 4 |
| **Key Feelings** |  |  |  |  |  |  |
| Anger | 3920 | 11.92 | 437 | 11 | 108 | 3 |
| Anxious | 4525 | 13.76 | 520 | 11 | 111 | 2 |
| Confident | 581 | 1.77 | 16 | 3 | 8 | 1 |
| Confusion | 6283 | 19.10 | 683 | 11 | 155 | 2 |
| Disconnected | 4295 | 13.06 | 721 | 17 | 200 | 5 |
| Elated | 86 | 0.26 | 5 | 6 | 1 | 1 |
| Emotional | 2893 | 8.80 | 551 | 19 | 177 | 6 |
| Exhausted | 1899 | 5.77 | 346 | 18 | 108 | 6 |
| Fear | 4897 | 14.89 | 818 | 17 | 217 | 4 |
| Frustration | 9851 | 29.95 | 909 | 9 | 193 | 2 |
| Grief/Loss | 3895 | 11.84 | 644 | 17 | 153 | 4 |
| Guilt | 2095 | 6.37 | 420 | 20 | 123 | 6 |
| Happy | 1023 | 3.11 | 5 | 0 | 22 | 1 |
| Hopeful | 3058 | 9.30 | 139 | 5 | 22 | 1 |
| Hopeless | 2545 | 7.74 | 786 | 31 | 322 | 13 |
| Numb | 377 | 1.15 | 112 | 30 | 64 | 17 |
| Rejected | 4374 | 13.30 | 691 | 16 | 209 | 5 |
| Sad | 7684 | 23.36 | 1372 | 18 | 373 | 5 |
| Shocked | 616 | 1.87 | 65 | 11 | 22 | 4 |
| Stressed | 9234 | 28.08 | 1179 | 13 | 307 | 3 |
| Stuck | 2705 | 8.22 | 414 | 15 | 91 | 3 |
| **LGBTQI** |  |  |  |  |  |  |
| Acceptance from others | 120 | 0.36 | 337 | 17 | 82 | 4 |
| Difficulty coming out | 58 | 0.18 | 292 | 5 | 50 | 1 |
| Information | 28 | 0.09 | 1 | 4 | 1 | 4 |
| Self-acceptance | 72 | 0.22 | 7 | 10 | 0 | 0 |
| **Loneliness Isolation** |  |  |  |  |  |  |
| Acceptance from others | 2030 | 6.17 | 337 | 17 | 82 | 4 |
| General Chat | 6117 | 18.60 | 292 | 5 | 50 | 1 |
| **Mental Health (Clinical Diagnosis)** | |  |  |  |  |  |
| Discrimination/Stigma | 76 | 0.23 | 28 | 37 | 11 | 14 |
| Issues with care provider | 289 | 0.88 | 110 | 38 | 51 | 18 |
| Issues with medication | 222 | 0.67 | 68 | 31 | 27 | 12 |
| Not coping | 899 | 2.73 | 412 | 46 | 174 | 19 |
| Self-managing symptoms with support | 2085 | 6.34 | 71 | 3 | 5 | 0 |
| Struggling to cope – moderate impact on QOL | 1864 | 5.67 | 317 | 17 | 31 | 2 |
| **Parenting Problems** |  |  |  |  |  |  |
| Care issues | 380 | 1.16 | 61 | 16 | 14 | 4 |
| Children | 606 | 1.84 | 93 | 15 | 18 | 3 |
| Co-parenting | 212 | 0.64 | 24 | 11 | 4 | 2 |
| Teens | 282 | 0.86 | 317 | 17 | 31 | 2 |
| **Physical Health** |  |  |  |  |  |  |
| Acute | 570 | 1.73 | 56 | 10 | 7 | 1 |
| Chronic | 1399 | 4.25 | 154 | 11 | 30 | 2 |
| **Poverty/Financial concerns** | |  |  |  |  |  |
| Accommodation/refuge | 258 | 0.78 | 52 | 20 | 12 | 5 |
| Bankruptcy/debt | 270 | 0.82 | 58 | 21 | 15 | 6 |
| Food/clothing | 177 | 0.54 | 38 | 21 | 8 | 5 |
| Homelessness | 131 | 0.40 | 35 | 27 | 14 | 11 |
| **Relationship Difficulties** |  |  |  |  |  |  |
| Issues with family | 3530 | 10.73 | 494 | 14 | 136 | 4 |
| Issues with neighbours etc | 1031 | 3.13 | 84 | 8 | 13 | 1 |
| Issues with partner/spouse | 2538 | 7.72 | 285 | 11 | 51 | 2 |
| Issues with peers | 1153 | 3.51 | 152 | 13 | 37 | 3 |
| Relationship breakup | 1350 | 4.10 | 241 | 18 | 67 | 5 |
| **Self-harm (urge to injury)** |  |  |  |  |  |  |
| Mild/moderate self-harm | 224 | 0.68 | 117 | 52 | 46 | 21 |
| Severe self-harm | 28 | 0.09 | 13 | 46 | 12 | 43 |
| Urge to self-harm | 423 | 1.29 | 175 | 41 | 67 | 5 |
| **Sexual Health Problems** |  |  |  |  |  |  |
| Abortion | 13 | 0.04 | 4 | 31 | 2 | 15 |
| Contraception | 12 | 0.04 | 2 | 17 | 0 | 0 |
| HIV/STD | 14 | 0.04 | 3 | 21 | 1 | 7 |
| Pregnancy | 31 | 0.09 | 5 | 16 | 2 | 6 |
| Puberty | 4 | 0.01 | 0 | 0 | 0 | 0 |
| Sexual Dysfunction | 21 | 0.06 | 1 | 5 | 9 | 7 |
| **Suicide** |  |  |  |  |  |  |
| Suicide in family | 160 | 0.49 | 52 | 33 | 22 | 14 |
| Suicide in peer | 123 | 0.37 | 31 | 25 | 9 | 7 |
| **Bullying** |  |  |  |  |  |  |
| Primarily at place of study | 146 | 0.44 | 30 | 21 | 4 | 3 |
| Primarily at the workplace | 127 | 0.39 | 22 | 17 | 6 | 5 |
| Primarily cyber bullying | 45 | 0.14 | 9 | 20 | 2 | 4 |
| Primarily SMS/TXT | 10 | 0.03 | 0 | 0 | 0 | 0 |
| Primarily street/community | 190 | 0.58 | 13 | 7 | 4 | 2 |
| **Violence** |  |  |  |  |  |  |
| Committed violent offence | 27 | 0.08 | 6 | 22 | 0 | 0 |
| Elder abuse victim | 25 | 0.08 | 2 | 8 | 0 | 0 |
| Harm to others | 42 | 0.13 | 9 | 21 | 1 | 2 |
| Intimidation/harassment | 126 | 0.38 | 12 | 10 | 1 | 1 |
| Trauma | 230 | 0.70 | 64 | 28 | 23 | 10 |
